# Machine learning-based triage model for elderly traumatic brain injury patients in Chinese emergency department

**DOI:** 10.3389/fneur.2026.1817051

**Published:** 2026-05-25

**Authors:** Yanya Lin, Chengda Lin, Jianhui Chen, Shijun Chen, Jianhuang Huang, Jianxiong Hu

**Affiliations:** 1 Department of Critical Care Medicine, Affiliated Hospital of Putian University, Putian, China; 2 Department of Neurosurgery, Putian 95 Hospital of China RongTong Medical and Health Group, Putian, China; 3 Department of Neurosurgery, Affiliated Hospital of Putian University, Putian, China

**Keywords:** clinical decision support, elderly, emergency department, machine learning, SHAP, traumatic brain injury, triage

## Abstract

**Background:**

Elderly traumatic brain injury (TBI) patients pose triage challenges in emergency departments due to complex physiology and comorbidities. Traditional methods often miss high-risk cases, necessitating accurate models to optimize ICU allocation.

**Methods:**

We developed an XGBoost-based triage model using data from 413 elderly TBI patients (aged ≥60 years) at Putian University Affiliated Hospital (2015–2024). Features included symptoms, CT hematoma density, contusion severity, age, anticoagulant use, and GCS scores. The target was ICU triage disposition within 48 h of ED presentation. Data were split (80:20) into training and testing sets. Class imbalance was handled within the training process using SMOTE, and model performance was assessed with 5-fold cross-validation using AUC, recall, precision, F1 score, and MCC. Because this was a prediction study rather than a causal inference analysis, all model outputs were interpreted as prediction of historical ICU triage disposition rather than proof of true ICU need. Complete-case analysis was used for model development after exclusion of records with missing key variables. SHAP analysis was used to improve interpretability.

**Results:**

The XGBoost model achieved an AUC of 0.93, recall of 0.9288, precision of 0.9274, F1 score of 0.9249, and MCC of 0.7241, outperforming the comparator models. Symptoms, CT hematoma density, and contusion severity were key predictors. Decision curve analysis suggested a higher theoretical net benefit than the “treat all” and “treat none” strategies across a clinically relevant threshold range; however, this finding should not be interpreted as a direct estimate of reduced unnecessary ICU admissions without prospective outcome-based validation.

**Conclusion:**

The XGBoost model provides an interpretable tool for predicting ICU triage disposition in elderly TBI patients and may support, rather than replace, emergency physician decision-making. Because the endpoint was a single-center process-of-care surrogate, further prospective and multicenter validation against patient-centered outcomes, including mortality, neurological deterioration, neurosurgical intervention, and functional status, is required.

## Introduction

With the intensification of global aging, the incidence of traumatic brain injury (TBI) among elderly populations has significantly increased (1). Elderly patients exhibit more complex post-TBI clinical courses and marked prognostic heterogeneity due to brain atrophy, increased vascular fragility, and multiple comorbidities (2, 3). In emergency department (ED) settings, rapidly and accurately deciding whether elderly TBI patients should be admitted to general wards or directly to intensive care units (ICU) poses a significant clinical challenge (4).

Traditional triage decisions mainly rely on physician experience, trauma scores (e.g., Glasgow Coma Scale [GCS], consciousness level), and vital signs. However, multiple studies indicate these methods have limited predictive power in elderly patients, potentially causing over- or under-treatment (5, 6). Elderly patients often demonstrate “pseudo-stability” with mild initial symptoms but rapid subsequent deterioration (7). In China, due to the low ICU bed ratio, precise triage models are essential to optimize resource allocation for elderly TBI patients (8). Therefore, developing an intelligent triage model integrating multidimensional data to identify covert high-risk individuals is of important clinical significance.

In recent years, machine learning (ML) techniques have shown notable advantages in trauma prognosis prediction and ICU admission determination. Models based on multimodal data from electronic medical records (EMR), laboratory indicators, and imaging features can surpass the linear constraints of traditional regression methods, achieving higher accuracy in risk stratification (9). For example, some researchers have developed ML algorithms to predict ICU admission in elderly trauma patients, significantly improving model accuracy and interpretability (6). Another study utilized deep learning to integrate cranial CT imaging features, enabling dynamic prediction of early neurological deterioration in TBI patients (10). However, ML research specifically addressing triage of elderly TBI patients in ED scenarios remains relatively scarce. Existing studies mainly focus on general trauma populations with single-modal data (e.g., only GCS or CT imaging), failing to fully consider elderly patients’ physiological characteristics, pharmacological interventions (e.g., anticoagulant use), and data constraints imposed by ED decision timeframes (11). Additionally, differences in resource allocation among institutions (e.g., general ward versus ICU bed ratios, stratified physician experience) further challenge clinical model implementation (2, 8).

This study aims to construct and validate an intelligent triage model suitable for elderly TBI patients based on real-world ED data, using historical ICU versus general ward triage disposition as the prediction target and exploring key features influencing model performance. Using ML methods, this study intends to provide an objective and quantifiable support tool for early management of elderly TBI patients, while explicitly recognizing that ICU admission is a process-of-care surrogate rather than a direct patient-centered outcome.

## Methods

### Data collection

**Figure 1:**
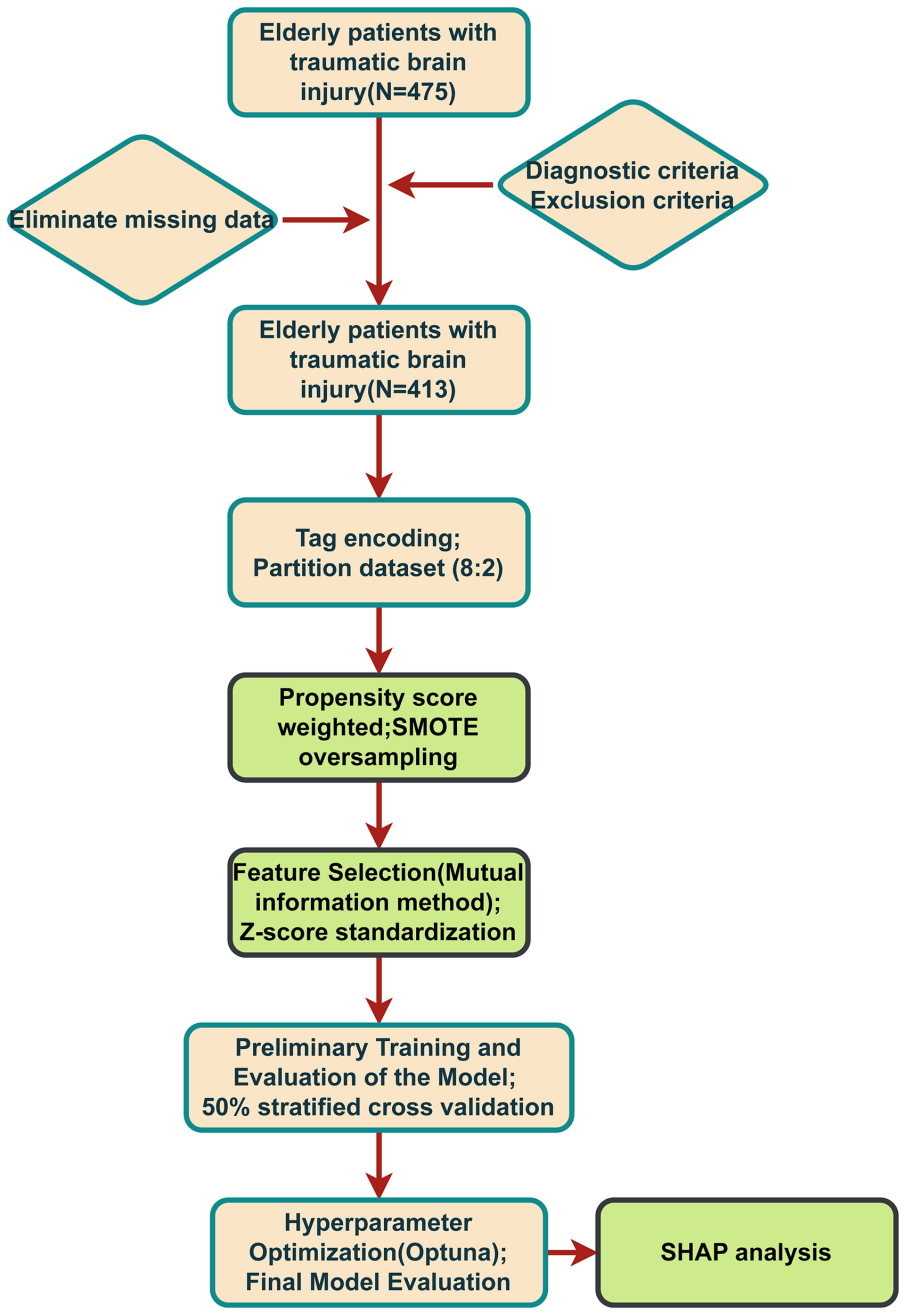
Technical workflow of the study.

This study employed a retrospective cohort design using data from elderly TBI patients admitted to Putian University Affiliated Hospital between January 2015 and December 2024. The initial dataset included 413 records. After complete-case filtering, 316 patients with non-missing values for all variables used in model development were retained for analysis. The dataset includes patients’ basic information, clinical features, laboratory results, and final triage outcomes (patient location in ICU or general ward within 48 h). Each sample contains multiple clinical features (e.g., age, sex, comorbidities, injury mechanisms, imaging and laboratory results), with the target variable defined as historical ICU triage disposition rather than confirmed biological necessity for ICU care. The study was approved by the Ethics Committee of Putian University Affiliated Hospital (ID: 202555-1). Figure 1 illustrates the study’s technical workflow.

Inclusion criteria: (1) age ≥ 60 years, based on epidemiologic characteristics of China’s elderly population (12); (2) confirmed diagnosis of traumatic brain injury in the ED, including but not limited to concussion, brain contusion, intracranial hematoma (subdural, epidural, intracerebral), or skull fracture confirmed by clinical evaluation and head CT imaging; (3) initial assessment and triage decision (ICU or general ward) completed within 48 h of ED admission (13, 14) to ensure model relevance to time-sensitive ED decisions; (4) data completeness with key predictors available in EMR, including symptoms (e.g., vomiting, impaired consciousness), CT hematoma density, brain contusion severity, age, anticoagulant/antiplatelet therapy history, and GCS.

Exclusion criteria: (1) non-traumatic brain lesions, such as ischemic or hemorrhagic stroke, brain tumors, epilepsy, or metabolic encephalopathies; (2) records with >20% missing key predictors (e.g., missing GCS score, CT imaging, or anticoagulant therapy history) to ensure training data quality; (3) transferred patients from other institutions prior to ED evaluation, as pre-transfer interventions may cause data inconsistency; (4) triage performed beyond 48 h after admission, to avoid confounding from long-term hospitalization events (e.g., infection, complications); (5) non-ED admissions (e.g., outpatient); (6) severe polytrauma cases (e.g., thoracoabdominal injury or long bone fractures with Injury Severity Score [ISS] > 15) to focus on isolated TBI triage prediction.

### Data preprocessing

Samples with missing values were removed to ensure data completeness. Specifically, complete-case analysis was performed and 97 records with missing data, all driven by missingness in Medical_history, were excluded before model development. All non-numeric variables were label-encoded using pandas’ factorize() for modeling compatibility. The dependent variable (y) was historical ICU triage disposition within 48 h after ED admission. Accordingly, the model was designed to learn institutional triage patterns under real-world practice conditions, and not to establish whether ICU admission was causally beneficial or universally appropriate for each patient.

### Dataset splitting and exploratory sample weighting

The complete dataset was randomly split 80:20 into training and test sets with random_state = 42. The training set was used for model development and feature selection, while the test set served as an independent validation set for final evaluation, strictly excluding any tuning or sampling to avoid data leakage. Because this was a prediction study rather than a causal inference analysis, ICU triage disposition was not treated as an intervention effect. In the original workflow, propensity-score-derived weights were explored only as a balancing strategy to reduce marked baseline heterogeneity between patients historically triaged to ICU and those triaged to the general ward. In the revised manuscript, we explicitly avoid language implying simulation of a randomized trial or correction of causal confounding, and we interpret any weighted results as exploratory and institution-specific rather than causal.

### Class imbalance handling: SMOTE oversampling

Given the low proportion of ICU admissions (positive class), SMOTE (Synthetic Minority Over-sampling Technique) was applied within training folds during 5-fold stratified cross-validation to generate synthetic minority samples, enhancing positive class recognition. SMOTE was strictly confined to training data folds, excluding validation and test sets to prevent information leakage and evaluation bias. Changes in sample sizes pre- and post-SMOTE were illustrated by histograms. Principal component analysis (PCA) reduced feature dimensionality to two components for visualizing original and synthetic sample distributions, supporting the reasonability of SMOTE-generated samples. In the revised manuscript, this step is presented strictly as a class-imbalance handling technique for predictive modeling, not as a substitute for clinical outcome validation.

### Feature selection

To reduce dimensionality, improve generalizability, and enhance interpretability, mutual information was first used to select the top 20 features most correlated with the target variable. Subsequently, L1-regularized logistic regression (C = 1.0) and XGBoost feature importance further refined the feature set to the final 10 key variables used for modeling. Selected features were standardized via Z-score scaling (mean = 0, variance = 1) to stabilize and accelerate training, especially for gradient-boosting and distance-based algorithms.

### Preliminary model training and evaluation

Eight representative classifiers were compared: Logistic Regression, Random Forest, Support Vector Machine (SVM), Gaussian Naive Bayes, Gradient Boosting, XGBoost, LightGBM, and CatBoost. Models were trained and evaluated using 5-fold stratified cross-validation to ensure robustness, maintaining consistent positive–negative ratios in each fold. Within each fold, SMOTE and feature selection were applied only to training subsets; validation subsets remained unaltered to prevent data leakage. Any propensity-score-derived sample weights, where explored, were used only in a sensitivity-oriented predictive context and were not interpreted causally. Evaluation metrics included AUC (Area Under the Receiver Operating Characteristic Curve), accuracy, F1-score, precision, recall, and Matthews correlation coefficient (MCC). Predictions, labels, and probabilities were saved for comprehensive analysis and visualization.

### Hyperparameter optimization

The top-performing XGBoost model underwent automated hyperparameter tuning via Optuna, maximizing 5-fold CV AUC. Parameters tuned included: n_estimators (100–500), max_depth (3–10), learning_rate (0.01–0.3), subsample (0.6–1.0), colsample_bytree (0.6–1.0), gamma (0–1), and min_child_weight (1–10), with random seed 42. The optimal parameter set was selected and the final model retrained on the full training dataset.

### Final model evaluation and validation

Comprehensive evaluation on the independent test set (or aggregated cross-validation results) focused on discrimination, calibration, clinical utility, and decision curve analysis (DCA). DCA was used only to describe theoretical net benefit across threshold probabilities, rather than to quantify actual reductions in unnecessary ICU admission. Classification metrics were summarized with bar and error bar plots presenting mean ± standard deviation.

### SHAP interpretability

SHapley Additive exPlanations (SHAP) were employed to interpret the model’s predictions by quantifying each feature’s contribution, revealing the model’s black-box behavior. SHAP summary plots and force plots were generated to elucidate individual patient predictions and overall feature impact on ICU triage decisions.

### Statistical analysis

All analyses were performed using Python’s scikit-learn library. Cross-validation assessed model generalizability. Final model performance was validated on an independent test set. Standardized calculation methods were used for all metrics.

## Results

### Demographics and baseline characteristics

A total of 413 elderly TBI patient records were initially available in the curated dataset. Under complete-case analysis, 97 records with missing values were excluded, leaving 316 patients for model development, including 275 in the general ward group and 41 in the ICU group. Table 1 summarizes demographics and baseline features comparing general ward and ICU groups in the analyzed cohort. The general ward group had significantly higher GCS, dementia prevalence, high-density hematoma rate, and female proportion, while age, anticoagulant/antiplatelet therapy, Rotterdam CT score, hematoma volume, and contusion severity were significantly lower than the ICU group (*p* < 0.05).

**Table 1:** Clinical characteristics of elderly TBI patients by ward type.

Variable	General ward	ICU	*p*
GCS	12.94 ± 1.84	11.10 ± 2.26	<0.001
Dementia	14%(Y)	4.5%(Y)	<0.001
Symptom	Headache (94.2%); vomit (3.6%); Neurological dysfunction (1.1%); convulsion (1.1%)	Headache (24.4%); vomit (41.5%); Neurological dysfunction (17.1%); convulsion (17.1%)	<0.001
Gender	Male (72.4%); female (27.6%)	Male (90.2%); female (9.8%)	0.01
Age, years	69.25 ± 6.95	73.00 ± 7.64	<0.001
Anticoagulant/antiplatelet therapy	0.04 ± 0.20	0.32 ± 0.47	<0.001
Primary diagnosis	Subdural Hematoma (87.3%); Epidural Hematoma (12.7%)	Subdural Hematoma (95.1%); Epidural Hematoma (4.9%)	0.14
Rotterdam CT	2.68 ± 0.47	2.93 ± 0.26	<0.001
Hematoma volume, cm^3^	8.53 ± 2.56	10.56 ± 3.85	<0.001
Location	Frontal (38.2%); parietal (23.6%); temporal (22.2%); occipital (16.0%)	Frontal (39.0%); parietal (26.8%); temporal (29.3%); occipital (4.9%)	0.27
CT density	High (88.4%); Low (11.6%)	Low (51.2%); high (48.8%)	<0.001
CCL*	Mild (52.7%); Moderate (27.3%); Severe (20.0%)	Mild (9.8%); Moderate (51.2%); Severe (39.0%)	<0.001
Midline shift	<0.5 cm (64.7%); >0.5 cm and <1 cm (2.5%); >1 cm (32.7%)	<0.5 cm (53.7%); >0.5 cm and <1 cm (4.9%); >1 cm (41.5%)	0.34
Trauma mechanism	Fall (50.2%); traffic accidents (24.4%); assault (10.5%); fall from height (14.9%)	Fall (36.6%); traffic accidents (34.1%); assault (19.5%); fall from height (9.8%)	0.12
Medical_history	DM (37.1%); Hypertension (36.4%); Both (26.5%)	DM (22.0%); Hypertension (41.5%); Both (36.6%)	0.14
Temperature, °C	36.94 ± 0.58	37.01 ± 0.56	0.48
Heart rate, bpm	94.50 ± 20.42	92.51 ± 17.70	0.56
SBP, mmHg	133.27 ± 19.13	136.78 ± 19.64	0.28
DBP, mmHg	77.86 ± 10.08	80.71 ± 9.00	0.09
Hb, g/L	115.92 ± 11.41	115.23 ± 11.67	0.72
WBC,×10^9^/L	17.03 ± 5.55	16.69 ± 5.41	0.71
NEUT,×10^9^/L	13.11 ± 5.97	12.76 ± 6.11	0.73
NEUTp	0.72 ± 0.13	0.73 ± 0.13	0.77
PLT,×10^9^/L	178.85 ± 23.72	175.44 ± 21.79	0.39
LYMPHp	12.09 ± 2.09	11.63 ± 2.21	0.19
MPV, fL	12.49 ± 1.36	12.36 ± 1.27	0.57
PDW	16.69 ± 2.69	16.80 ± 2.77	0.82
ALT, U/L	23.39 ± 6.14	24.36 ± 6.58	0.35
AST, U/L	30.96 ± 5.95	32.15 ± 5.22	0.22
A/G	1.64 ± 0.30	1.60 ± 0.29	0.42
Cr, μmol/L	67.78 ± 9.87	68.84 ± 8.36	0.51
BUN, mmol/L	5.13 ± 1.79	5.61 ± 1.96	0.11
Na, mmol/L	139.66 ± 10.12	133.97 ± 9.89	0.00
K, mmol/L	4.51 ± 0.85	4.70 ± 0.90	0.20
Ca, mmol/L	1.52 ± 0.30	1.52 ± 0.32	0.95
Cl, mmol/L	103.02 ± 7.57	103.86 ± 7.15	0.50
PT,s	13.29 ± 0.61	13.30 ± 0.62	0.93
APTT,s	34.42 ± 1.58	34.48 ± 1.59	0.82
FIB, g/L	3.65 ± 0.35	3.64 ± 0.36	0.95
D-Dimer, mg/L	1.95 ± 0.34	1.96 ± 0.33	0.91
TT,s	18.18 ± 0.78	18.20 ± 0.79	0.90
Glu, mmol/L	9.20 ± 0.79	9.37 ± 0.80	0.18

*CLL, Cerebral contusion and laceration.

### Propensity score matching and sample weighting

**Figure 2:**
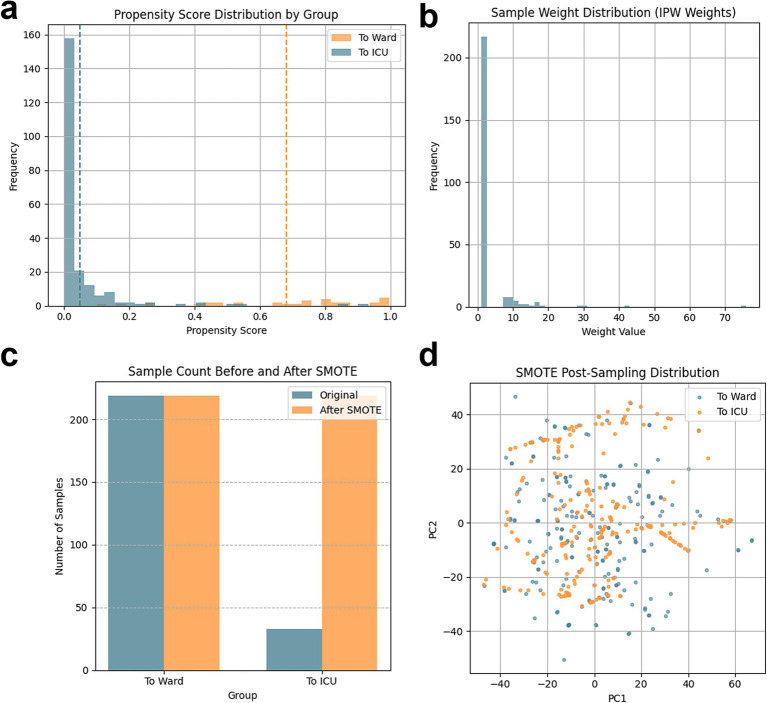
Modeling strategies for class imbalance and exploratory balancing diagnostics. **(a)** Propensity score distributions for ICU (*n* = 33) and general ward (*n* = 219) groups, showing separation between historically observed triage groups. **(b)** Distribution of propensity-score-derived sample weights explored during model development; these weights are presented as balancing diagnostics rather than causal adjustment. **(c)** Comparison of sample sizes before and after SMOTE oversampling in the training set, highlighting a substantial increase in minority class (ICU) samples to improve class balance. **(d)** SMOTE-generated synthetic sample distribution visualized via two-dimensional principal component analysis, demonstrating that synthetic samples cluster near original minority class points without introducing obvious noise.

The ICU-to-general ward ratio was approximately 1:6.25, indicating substantial class imbalance. Propensity-score-derived weights showed clear separation between historically observed triage groups and were explored as balancing diagnostics during model development (Figures 2a,b). However, these distributions should not be interpreted as evidence of true ICU need or as a causal adjustment for treatment effects. SMOTE oversampling markedly increased minority class samples in training folds (Figure 2c). PCA visualizations confirmed that synthetic samples generated by SMOTE closely followed the original minority class distribution without obvious noise inflation (Figure 2d).

### Feature selection and standardization

**Figure 3:**
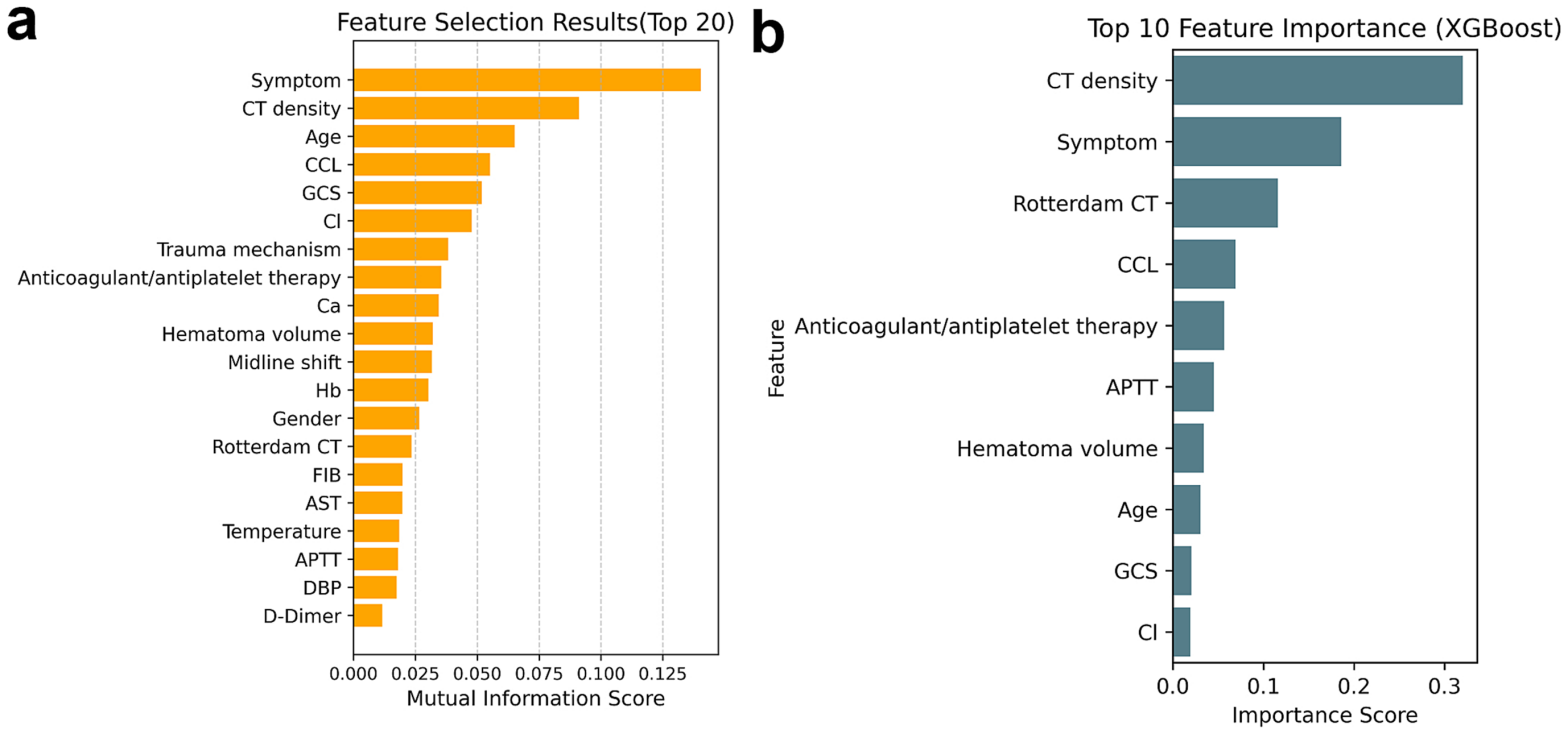
Feature selection and XGBoost feature importance analysis. **(a)** Feature selection identified the top 20 features most associated with ICU admission (highlighted in orange), including symptoms, CT density, age, cerebral contusion and laceration (CLL), and Glasgow Coma Scale (GCS). **(b)** Top 10 feature importance scores from the XGBoost model, presented as a bar chart.

Mutual information selected 20 features most correlated with ICU admission (Figure 3a), including symptoms, CT hematoma density, age, brain contusion severity, and GCS. StandardScaler normalized these features with means near zero and standard deviations near one. XGBoost feature importance ranked the top 10 features (Figure 3b).

### Preliminary model evaluation

**Figure 4:**
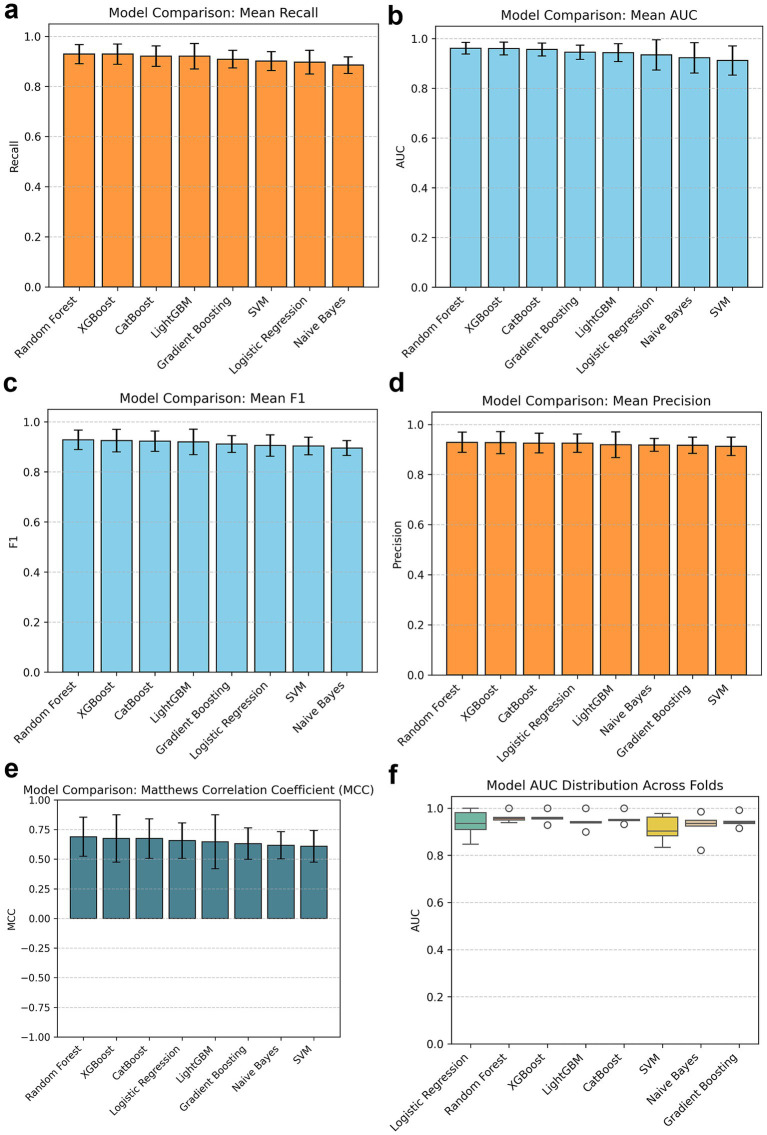
Performance comparison of triage models for elderly traumatic brain injury patients. **(a)** Recall rates for eight models detecting ICU-need patients, with XGBoost achieving the highest recall (0.9288), identifying ~93% of high-risk cases and minimizing missed diagnoses. **(b)** AUC comparison for distinguishing ICU vs. non-ICU patients, where XGBoost attains the highest average AUC (0.9602), indicating superior risk stratification. **(c)** F1 score comparison reflecting balance between precision and recall; XGBoost leads with 0.9249, maintaining high recall while controlling false positives. **(d)** Precision rates showing XGBoost’s high accuracy (0.9274) and low false positive rate in identifying ICU candidates. **(e)** Overall classification performance measured by Matthews correlation coefficient (MCC), with XGBoost achieving the highest average MCC (0.7241). **(f)** Stability assessment based on AUC standard deviation, where XGBoost demonstrates the lowest variability, indicating reliable clinical applicability.

Among eight ML models, XGBoost achieved the highest recall (0.9288; 95% CI: 0.90–0.95), significantly outperforming other models (Figure 4a). This indicates that approximately 93% of patients historically triaged to ICU in our cohort were correctly identified by the model, thereby reducing the risk of under-predicting ICU triage disposition within this single-center dataset. CatBoost and LightGBM followed with recalls of 0.9209 and 0.9187, respectively. Traditional models like Naive Bayes (0.8969) and Logistic Regression (0.9012) had lower recall, raising concern for missed high-risk patterns under the observed institutional triage practice. XGBoost maintained superior overall discrimination (AUC 0.9602; 95% CI: 0.94–0.98) and precision (0.9274), balancing sensitivity and specificity effectively (Figures 4b–d). Its MCC was highest (0.7241; 95% CI: 0.70–0.75) (Figure 4e), with minimal AUC variability across folds (Figure 4f), confirming robustness.

### Optimal model training and value

**Figure 5:**
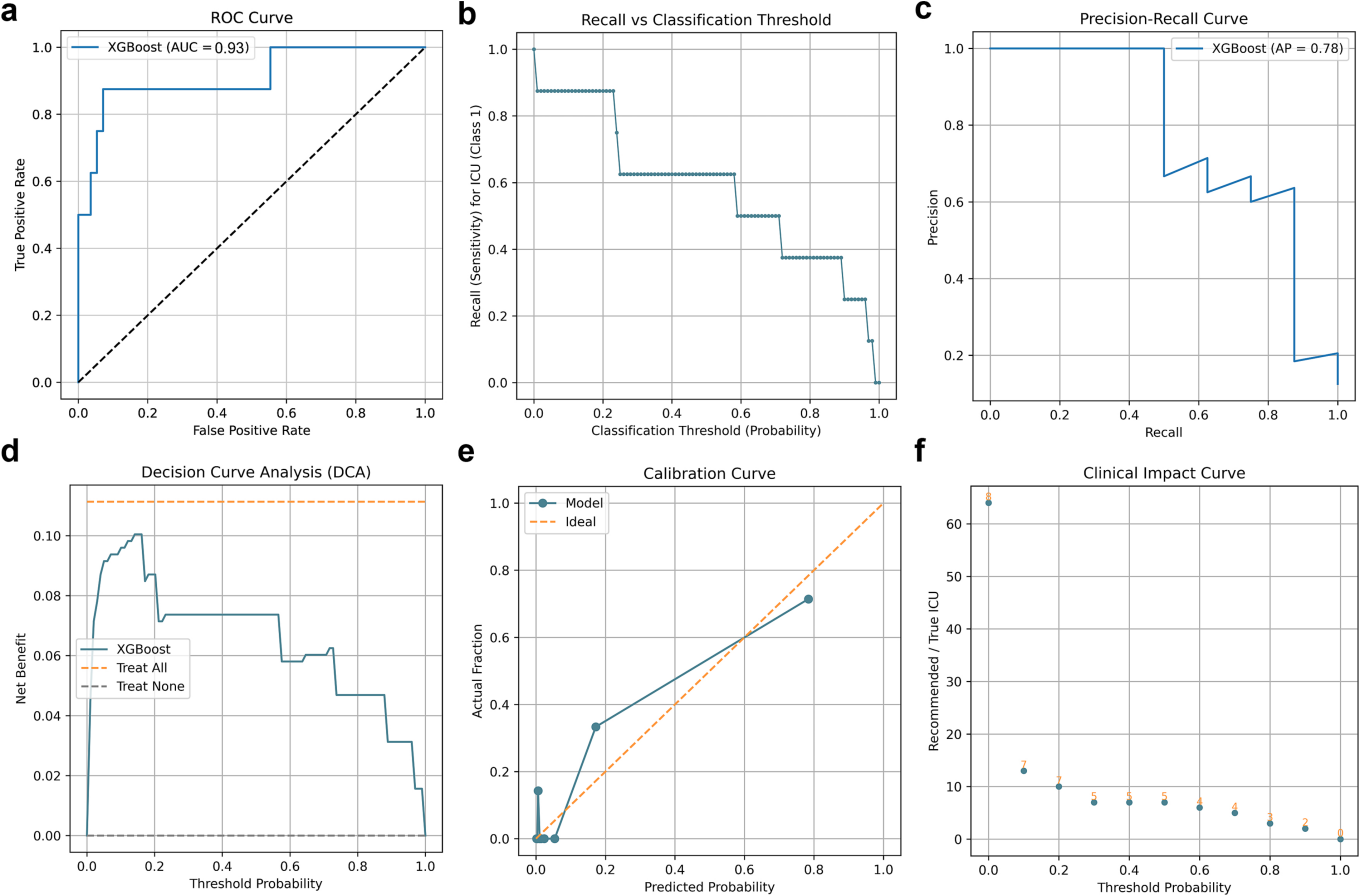
Performance evaluation of the XGBoost model for ICU triage disposition in elderly traumatic brain injury patients. **(a)** ROC curve with an AUC of 0.93, demonstrating strong prediction of ICU triage disposition. **(b)** Recall versus classification threshold: ~0.85 at low threshold (0.2) and ~0.38 at high threshold (0.8). **(c)** Precision-recall curve with average precision (AP) of 0.78. **(d)** Decision curve analysis (DCA) showing theoretical net benefit of the model relative to “Treat All” and “Treat None” strategies across thresholds. **(e)** Calibration curve illustrating alignment between predicted ICU probabilities and observed ICU triage disposition rates. **(f)** Clinical impact curve depicting the number of patients recommended for ICU triage versus observed ICU triage cases across varying probability thresholds; these curves should not be interpreted as direct evidence of reduced unnecessary ICU use without prospective outcome validation.

After hyperparameter tuning, the XGBoost model achieved an AUC of 0.93 and showed strong performance in predicting ICU triage disposition among elderly TBI patients (Figure 5a). Recall varied with classification threshold—from approximately 0.85 at 0.2 threshold to 0.38 at 0.8 (Figure 5b). The model’s average precision (AP) was 0.78 (Figure 5c). Decision curve analysis suggested that the model may provide a higher theoretical net benefit than “treat all” and “treat none” strategies across a range of threshold probabilities (Figure 5d). Calibration curves indicated generally optimistic yet acceptable probability predictions across risk strata (Figure 5e). Clinical impact curves illustrated the relationship between predicted ICU triage recommendations and observed ICU triage disposition across thresholds, which may help inform threshold selection but do not prove reductions in unnecessary ICU use without prospective outcome-based validation (Figure 5f).

### SHAP interpretation

**Figure 6:**
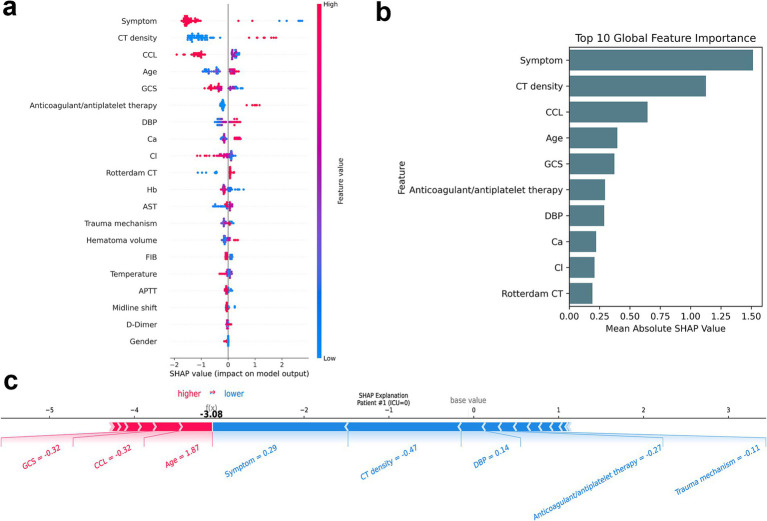
SHAP analysis of XGBoost model predicting ICU need in elderly traumatic brain injury patients. **(a)** Distribution of SHAP values for key features. **(b)** Feature importance ranking based on mean absolute SHAP values. **(c)** Individual SHAP force plot illustrating feature contributions. CLL, cerebral contusion and laceration.

SHAP analysis elucidated key feature contributions to ICU admission prediction (Figure 6a). Top predictors included symptoms, CT hematoma density, brain contusion severity, age, anticoagulant/antiplatelet therapy, GCS, and diastolic blood pressure (DBP). Symptoms, CT hematoma density, and contusion severity showed broad, substantial SHAP value ranges, indicating dominant influence on ICU demand prediction (Figure 6b). Individual SHAP force plots demonstrated how mild symptoms, low inflammatory markers, and stable vital signs (e.g., normal temperature, blood pressure) led to low predicted ICU admission probability (f(*x*) = −3.08) (Figure 6c).

## Discussion

Based on real-world ED data, this study developed and validated an intelligent triage model for elderly TBI patients. The XGBoost model achieved an AUC of 0.93 and a recall of 0.9288 on an independent test set, outperforming seven other classifiers in predicting historical ICU triage disposition while maintaining high precision (0.9274) and MCC (0.7241). Key predictors included symptoms, CT hematoma density, contusion severity, age, anticoagulant/antiplatelet therapy, and GCS. SHAP analyses confirmed that high-risk features such as low-density CT hematoma and severe brain contusion were strongly associated with higher model-predicted ICU triage probability, providing an interpretable description of the patterns underlying past triage decisions. Importantly, these findings should be interpreted as prediction of a process-of-care surrogate from a single center, rather than confirmation of true ICU need or benefit from ICU admission.

### Comparison with existing literature

Our findings align with and extend recent ML applications in elderly trauma triage. Si et al. reported an XGBoost model predicting elderly TBI mortality (AUC = 0.91), identifying age and GCS as primary features (15). Wang et al. (16) used Random Forest to model consciousness disorders in ED brain trauma, highlighting advanced age and low GCS. These studies lacked CT hematoma density and symptom inclusion, limiting specificity. Similarly, Zhang et al. (17) employed LightGBM for TBI in-hospital outcomes (AUC ~ 0.88, recall ~0.85), using SHAP for interpretation but omitted anticoagulant use—a key elderly factor ranked sixth in our SHAP analysis. Our higher recall (0.9288) likely stems from multimodal data integration (EMR, labs, CT) and richer characterization of elderly-specific risk factors (18). GCS alone shows limited predictive calibration in elderly TBI due to pseudo-stability masking deterioration (19). In the revised manuscript, we have moderated our interpretation of decision curve analysis: DCA supports potential theoretical net benefit in this dataset, but it does not by itself quantify preventable ICU admissions or prove improved patient outcomes.

### Clinical and research implications

Clinically, our model should be considered an adjunctive decision-support tool rather than an autonomous triage rule. It may help ED physicians identify elderly TBI patients whose presentation resembles previously ICU-triaged cases, especially when symptoms and CT hematoma density suggest fragile stability (20). Integration into EMR systems could standardize risk communication and support secondary review of borderline cases (21). However, the model should not be used to directly reassign a fixed proportion of patients away from ICU without prospective validation against outcomes such as mortality, neurological deterioration, need for neurosurgical intervention, and short-term functional status. The top 10 features are obtainable within 30 min, supporting practical deployment in resource-constrained ED environments.

### Strengths and limitations

This study has several strengths. It is, to our knowledge, among the first ML studies focused specifically on ED triage disposition for elderly TBI patients using real-world data from Putian University Affiliated Hospital. Five-fold stratified cross-validation and independent testing minimized overfitting. SHAP improved transparency by enabling individualized explanation of model predictions and may facilitate an “AI-assisted plus human oversight” workflow (22). Nevertheless, the clinical meaning of the model must be interpreted cautiously because the predicted endpoint was ICU triage disposition rather than a patient-centered outcome.

The study also has important limitations. First, ICU admission is a process-of-care surrogate determined by local clinicians and local resource constraints; therefore, the model may learn historical institutional practice patterns, including potential over-triage, under-triage, and inconsistency, rather than universally valid ICU need. Second, because this was a single-center retrospective study, external validity remains uncertain and multicenter validation is required. Third, although propensity-score-derived weighting was explored during model development, such weighting does not create a causal design in this setting, and combining weighting with SMOTE may introduce additional modeling dependence; accordingly, we have revised the manuscript to avoid causal language and to treat these steps as exploratory. Fourth, complete-case analysis excluded 97 of 413 records because of missing data, primarily missing Medical_history, which may have introduced selection bias and reduced representativeness; future studies should consider multiple imputation (MICE) (23, 24) or prospectively improved data capture. Finally, prospective validation should use outcomes such as in-hospital mortality, neurological deterioration after ED triage, neurosurgical intervention, mechanical ventilation, ICU-specific therapies, discharge disposition, and 30/90-day functional status to determine whether the model improves patient-centered care.

## Conclusion

This XGBoost model offers an interpretable approach for predicting ICU triage disposition in elderly TBI patients and may serve as a supportive tool in emergency settings. Its current scope is hypothesis-generating and operational rather than definitive. Prospective, multicenter, outcome-based validation is necessary before using the model to change ICU allocation policy.

## Data Availability

The raw data supporting the conclusions of this article will be made available by the authors, without undue reservation.
